# Edge computing for Vehicle to Everything: a short review

**DOI:** 10.12688/f1000research.73269.1

**Published:** 2021-11-01

**Authors:** Mohd. Fikri Azli Abdullah, Sumendra Yogarayan, Siti Fatimah Abdul Razak, Afizan Azman, Anang Hudaya Muhamad Amin, Mazrah Salleh

**Affiliations:** 1 Faculty of Information Science and Technology, Multimedia University, Ayer Keroh, Melaka, 75450, Malaysia; 2 Research and Innovation, Kolej Universiti Islam Melaka, Melaka, 78200, Malaysia; 3 Faculty of Computer, Information Science and Applied Media, Higher Colleges of Technology, Dubai, United Arab Emirates; 4 Civil Aero Data and Information, Rolls-Royce, Derby, England, UK

**Keywords:** V2X, Edge Computing, Review

## Abstract

Vehicle to Everything (V2X) communications and services have sparked considerable interest as a potential component of future Intelligent Transportation Systems. V2X serves to organise communication and interaction between vehicle to vehicle (V2V), vehicle to infrastructure (V2I), vehicle to pedestrians (V2P), and vehicle to networks (V2N). However, having multiple communication channels can generate a vast amount of data for processing and distribution. In addition, V2X services may be subject to performance requirements relating to dynamic handover and low latency communication channels. Good throughput, lower delay, and reliable packet delivery are the core requirements for V2X services.  Edge Computing (EC) may be a feasible option to address the challenge of dynamic handover and low latency to allow V2X information to be transmitted across vehicles. Currently, existing comparative studies do not cover the applicability of EC for V2X. This review explores EC approaches to determine the relevance for V2X communication and services. EC allows devices to carry out part or all of the data processing at the point where data is collected. The emphasis of this review is on several methods identified in the literature for implementing effective EC. We describe each method individually and compare them according to their applicability. The findings of this work indicate that most methods can simulate the EC positioning under predefined scenarios. These include the use of Mobile Edge Computing, Cloudlet, and Fog Computing. However, since most studies are carried out using simulation tools, there is a potential limitation in that crucial data in the search for EC positioning may be overlooked and ignored for bandwidth reduction. The EC approaches considered in this work are limited to the literature on the successful implementation of V2X communication and services. The outcome of this work could considerably help other researchers better characterise EC applicability for V2X communications and services.

## Introduction

**Figure 1.**
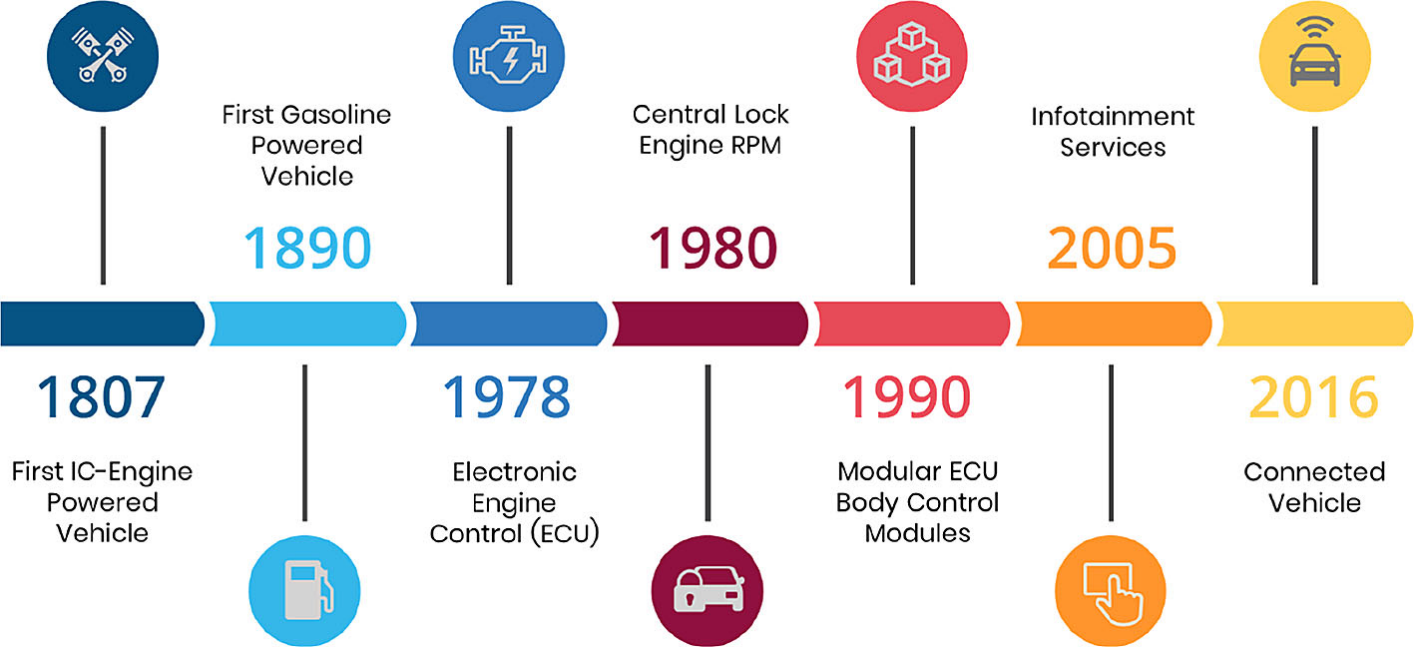
Evolution of driver and passenger interactions.

The automobile industry is changing in various ways, and this provides a chance to address potential transportation-related difficulties. This includes transitioning a traditional independent network to a connected network within and outside the vehicle. ^1^ The evolution of drivers' and passengers' involvement with vehicles has been evolving both in technology and style. ^2^ Figure 1 depicts the evolution of the interaction between drivers and passengers from 1807 to the present.

Almost everyone is connected to the Internet, with around six connected devices per person and hundreds of new connections created each second, resulting in billions of connected ecosystems. ^3^ Furthermore, research has projected that by 2025, connected vehicles will produce over 200 petabytes of data, with at least four terabytes of data generated continuously. This would increase the number of connected vehicles on roads by approximately four hundred million. ^4^ A connected vehicle is one that is equipped with both Internet and wireless LAN connectivity, allowing data to be transmitted between devices both inside and outside of the vehicle. The Internet of Vehicles or Vehicle to Everything (V2X) is a common network for connected vehicles. ^5^^,^^6^ However, the most challenging problem is efficiently processing and sending enormous data over communication networks.

The difficulty is not just handling data produced by these connected vehicles that are constantly exposed but also maintaining security, deployment, and performance. ^7^^,^^8^ Therefore, the potential of edge computing (EC) for V2X can play a prominent part. EC is a distributed computer system that carries out computational tasks (such as collecting and analysing data) on a device, particularly a vehicle. In turn, this reduces the transmission of data from the cloud back and forth. ^9^^,^^10^ This review examines EC, in particular for V2X. We discuss the background of automotive evolution, V2X and EC; prior research on the applicability of EC for V2X; the potential challenges of applying EC to the V2X scenario; and the path for the future.

## Automotive evolution, V2X, and EC

V2X communication is a crucial component of current intelligent transportation systems (ITS). For example, V2X provides drivers with information about road hazards that they may overlook. ^11^ In addition, V2X allows communication between a vehicle and anything that might impact the environment, including the surrounding infrastructure such as traffic lights (infrastructure) and even smartphones (pedestrian), enabling communication between vehicles and pedestrians holding a smartphone. ^12^ With the technology progressing globally, it is just a matter of time until it is widely adopted and deployed. ^13^

EC refers to a technology that allows network-level processing, downstream data for cloud services, and upstream data for IoT service support. ^14^ The term "edge" refers to any computer device located in the area between data sources and the cloud. EC is more suitable for applications that require rapid and consistent response times. ^15^ V2X is an example, as computing at the edge can reduce data transfer, decreasing reaction times. ^16^ For example, when driving, the vehicle captures data via movement, speed, and other sensors, then analyses them to ensure safety and convenience.

For V2X, real-time situational awareness is crucial, particularly on crucial route segments (e.g., an accident is detected by another vehicle on a particular road). Additionally, a backend server will have to provide high-definition local maps. Leveraging local maps and situational awareness is not just about providing data about road traffic conditions. It should also be extended to occurrences where local data must be aggregated in real-time and distributed to drivers on the road through road side units (RSUs). Road users may build and maintain real-time situational awareness using broadcast information from neighbour vehicles as an alternative to EC. Therefore, EC deployment enables shifting such activities to the network edge by combining data from many sources and efficiently broadcasting a huge amount of data to many drivers locally.

## Applicability of EC for V2X

EC applies to a wide range of uses, from sensor applications (e.g., predictive vehicle maintenance) to the end-user experience (e.g., collision prevention warning). EC has been discussed previously from the perspective of V2X communication applicability. In 2020, Moubayed *et al.* described an Optimum V2X Service Placement (OVSP) as a binary integer issue in a linear edge context. ^17^ The authors approached this problem using a low-complexity greedy heuristic technique (G-VSPA). Extensive simulations showed that the OVSP model provides satisfactory results when sensitive services to delays are on the edge and tolerant services to delays are at the core of the process. Furthermore, the proposed algorithm provides near-optimal performance with minimal complexity.

In the same year, Shaer *et al*. addressed the efficient deployment of V2X essential services, including various V2X applications in the EC environment. ^18^ The authors devised an optimisation method for minimising E2E latency in multi-component V2V systems under different traffic situations. The findings indicate that the methodology guarantees an adequate level of service and surpasses solutions developed in earlier studies using realistic scenarios. Additionally, Belogaev *et al*. investigated task offloading that minimises operating costs while adhering to the latency constraints imposed by different V2X applications, given the network architecture and resource allocation. ^20^ The authors designed a new CHAT algorithm based on linear programming and incorporated a greedy algorithm. In terms of total energy usage, the suggested method was compared with previous studies proposed algorithms. The assessment demonstrates that the proposed method considerably decreases energy usage while meeting the varied needs of V2X applications in all evaluated cases.

Lee *et al.* described an EC approach for minimising trip time at interconnected junctions. ^19^ The authors suggested a paradigm in which each RSU determines junction scheduling while the vehicles select their travel trajectory through dynamic control. Based on simulation results for optimum scheduling of linked junctions, the proposed framework significantly reduced overall travel time by up to 14.3%. Grammarikos and Cottis investigated the benefits of mobile edge computing (MEC) adopting V2X services linked to traffic efficiency and road safety. ^21^ A simulation model that represented a long-term evolution (LTE) system with basic MEC capabilities, such as packet routing, was investigated in this work to evaluate the applicability of their findings. The presented approach evaluated the packet delivery ratio and packet loss for applications, such as telemetry and emergency message delivery, respectively. While LTE can transmit traffic data to vehicles in a short amount of time, the simulation results revealed that severe congestion in the backhaul and core networks could result in unexpected packet losses, which could be prevented by the processing capabilities of a MEC server.

In addition, Napolitano *et al.* proposed a fully compatible design and implementation of a vulnerable road users (VRU) warning system, as well as an experimental assessment of the system using MEC- and cloud-based architectures. ^22^ The authors developed a strategy that would enable road users to communicate information regarding the existence of neighbouring entities in the event of a difficult circumstance (e.g., road accident). This is accomplished by using an architecture that consists of a user-facing Android application and a MEC-based application [cooperative awareness messages (CAM)]. The E2E latency demonstrated a substantial result when visualising the entities engaged between the VRUs application and the CAM server using a preliminary performance measurement. Additionally, Emara *et al.* focused on the case of VRU, examining the safe interaction of vehicles with road users such as motorcyclists and pedestrians. ^23^ The authors aimed to describe latency improvements using MEC systems through periodic CAM. Extensive simulation results indicated that installing MEC infrastructure may substantially decrease the communication latency. Additionally, Sabella *et al.* suggested a hierarchical MEC architecture for adaptive video streaming in V2X applications. ^25^ The authors described the acquisition of real-time channel data by local agents stationed at the evolved NodeB (eNB). This information is then communicated to a MEC platform, which automatically changes the video stream's quality to match the channel's conditions. Within a virtualized network context, the authors tested and evaluated a conceptual demonstration of radio-aware video optimization. The results demonstrated that the proposed architecture enhanced the user experience by boosting downlink and uplink speeds and reducing delay.

Bissmeyer *et al*. introduced a network framework that ensures V2X information and data exchange in a MEC-based multi-access technology environment. ^24^ The authors designed a framework for the integrity of the message, sender authorisation and authentication, and replay detection. This approach is achieved through digital signatures, an authorisation certificate, and public and private key infrastructure. MEC offers local processing capabilities for the exchange of event-driven V2X encrypted messages within the framework. In addition, Balid *et al.* demonstrated MEC traffic management methods for real-time traffic monitoring. ^26^ The authors developed and deployed a cost-effective wireless sensor traffic monitoring system for highway and roadside traffic. The sensor achieved an acceptable level of accuracy in terms of detection, speed prediction, and vehicle categorisation.

## Challenges of V2X and EC

### Security

At the edge of a network, privacy and security protection are critical services to provide. ^27^^,^^28^ If the vehicle is equipped with IoT, it can collect sensitive data from sense data. ^29^ Several ITS implementations would need drivers to grant access to sensitive, confidential data to untrusted vehicles attempting to join as edges in the context of smart cities. ^30^ Together with data segregation techniques, effective trust management systems may considerably increase edge security. ^31^ According to El-Sayed & Chaqfeh, ^31^ although minimal research has been conducted on assuring secure collaboration in an EC scenario, the study does not explicitly address V2X issues.

### Deployments

The positioning of edge devices in an urban environment is based on static and dynamic features. ^32^ Edge nodes may need MEC servers with fixed RSUs or unmanned aerial vehicles (UAV). ^33^ Many possible ITS applications may be facilitated by autonomous UAVs, improving traffic safety and transportation quality of life. ^34^ Nevertheless, specific issues must be addressed, such as limited energy, processing ability, and signal transmission range. ^35^ Given the technological developments such as sensor-based street lights or smart toll booths over the past few decades, the limitations on UAV usage will likely be overcome eventually.

### Performances

Each second counts when you're behind the wheel of a vehicle. As a result, vehicles would continuously upload the data collected by their local sensors to the closest edge device. ^36^^,^^37^ Hence, energy and power consumption at the edge should be considered to avoid service disruptions and quality of service (QoS) loss. ^38^^,^^39^ Furthermore, various situations need substantial QoS improvement to cope with occasional high traffic loads like severe traffic congestion, unpredicted weather conditions, or unexpected road construction works. ^40^ Therefore, further research is necessary to enhance and manage QoS in the V2X context considering a heterogeneous edge-based environment.

## Conclusions

EC adoption is growing in the automotive industry, and ITS, particularly V2X, will certainly change various economic sectors and significantly influence our everyday lives. Despite this, multiple different challenges are limiting its wide implementation. The increasing number of sensors in connected vehicles and roads creates a large data processing and storage issue. This requires new service platforms with strong processing, reliable storage, and real-time communication. EC is indeed a promising way to decrease latency and bring data closer to vehicles and resources. In the future, we will work on a comprehensive middleware solution for V2X communication. In many V2X scenarios, data transmitted between users and network infrastructure is localised and does not need remote access to centralised data centres. Using EC may substantially improve the performance of supporting various applications of V2X. The availability of network resources, storage, and computation near the network edge make EC an ideal option for V2X delay-sensitive applications.

## Data availability

No data is associated with this article.

## References

[1] Kirkland G : How new technologies have changed the automotive industry. 2019. Reference Source

[2] Shurpali S : Role of Edge Computing in Connected and Autonomous Vehicles. 2020. Reference Source

[3] Statista: Number of Internet of things (IoT) connected devices worldwide in 2018, 2025 and 2030. 2019. Reference Source

[4] Pati VP : Edge Insights for Superior Autonomous Vehicle Experience. 2020. Reference Source

[5] Uhlemann E : Introducing connected vehicles [connected vehicles]. *IEEE Vehicular Technology Magazine.* 2015;10(1):23–31. 10.1109/MVT.2015.2390920

[6] Coppola R Morisio M : Connected car: technologies, issues, future trends. *ACM Computing Surveys (CSUR).* 2016;49(3):1–36. 10.1145/2971482

[7] Guerrero-Ibáñez J Zeadally S Contreras-Castillo J : Sensor technologies for intelligent transportation systems. *Sensors.* 2018;18(4):1212. 10.3390/s18041212 29659524 PMC5948625

[8] Giust F Sciancalepore V Sabella D : Multi-access edge computing: The driver behind the wheel of 5G-connected cars. *IEEE Communications Standards Magazine.* 2018;2(3):66–73. 10.1109/MCOMSTD.2018.1800013

[9] Ai Y Peng M Zhang K : Edge computing technologies for Internet of Things: a primer. *Digital Communications and Networks.* 2018;4(2):77–86. 10.1016/j.dcan.2017.07.001

[10] Yousefpour A Fung C Nguyen T : All one needs to know about fog computing and related edge computing paradigms: A complete survey. *J. Syst. Archit.* 2019;98:289–330. 10.1016/j.sysarc.2019.02.009

[11] Kiela K Barzdenas V Jurgo M : Review of V2X–IoT standards and frameworks for ITS applications. *App. Sci.* 2020;10(12):4314. 10.3390/app10124314

[12] Naranjo JE Jiménez F Anaya JJ : Application of vehicle to another entity (V2X) communications for motorcycle crash avoidance. *J. Intell. Transp. Syst.* 2017;21(4):285–295. 10.1080/15472450.2016.1247703

[13] Ahlborn B : Five Reasons Why We Benefit from V2X. 2016. Reference Source

[14] Sittón-Candanedo I Corchado JM : An Edge Computing Tutorial. *Orien. J. Com. Sci. Techno.* 2019;12(2):34–38. 10.13005/ojcst12.02.02

[15] Shi W Dustdar S : The promise of edge computing. *Computer.* 2016;49(5):78–81. 10.1109/MC.2016.145

[16] Weisong S Xingzhou Z Yifan W : Edge computing: state-of-the-art and future directions. *J. Com. Res. Devel.* 2019;56(1):69.

[17] Moubayed A Shami A Heidari P : Edge-enabled V2X service placement for intelligent transportation systems. *IEEE Transactions on Mobile Computing.* 2020;20:1380–1392. 10.1109/TMC.2020.2965929

[18] Shaer I Haque A Shami A : Multi-Component V2X Applications Placement in Edge Computing Environment. *ICC 2020-2020 IEEE International Conference on Communications (ICC).* 2020; (pp.1–6).IEEE.

[19] Lee G Guo J Kim KJ : Edge Computing for Interconnected Intersections in Internet of Vehicles. *2020 IEEE Intelligent Vehicles Symposium (IV).* 2020; (pp.480–486). IEEE.

[20] Belogaev A Elokhin A Krasilov A : Cost-effective V2X task offloading in MEC-assisted intelligent transportation systems. *IEEE Access.* 2020;8:169010–169023. 10.1109/ACCESS.2020.3023263

[21] Grammatikos PV Cottis PG : A Mobile Edge Computing Approach for Vehicle to Everything Communications. *Communications and Network.* 2019;11(3):65–81. 10.4236/cn.2019.113006

[22] Napolitano A Cecchetti G Giannone F : Implementation of a MEC-based vulnerable road user warning system. *2019 AEIT International Conference of Electrical and Electronic Technologies for Automotive (AEIT AUTOMOTIVE).* 2019; (pp.1–6). IEEE.

[23] Emara M Filippou MC Sabella D : MEC-assisted end-to-end latency evaluations for C-V2X communications. *2018 European conference on networks and communications (EuCNC).* 2018; (pp.1–9). IEEE.

[24] Bissmeyer N Dam JF van Zimmermann C : Security in hybrid vehicular communication based on its-g5, lte-v, and mobile edge computing. *AmE 2018-Automotive meets Electronics; 9th GMM-Symposium.* 2018; (pp.1–6). VDE.

[25] Sabella D Nikaein N Huang A : A hierarchical MEC architecture: Experimenting the RAVEN use-case. *2018 IEEE 87th Vehicular Technology Conference (VTC Spring).* 2018; (pp.1–5). IEEE.

[26] Balid W Tafish H Refai HH : Intelligent vehicle counting and classification sensor for real-time traffic surveillance. *IEEE Transactions on Intelligent Transportation Systems.* 2017;19(6):1784–1794. 10.1109/TITS.2017.2741507

[27] Zhong S Zhong H Huang X : *Security and Privacy for Next-Generation Wireless Networks.* Springer International Publishing;2019.

[28] Zhang J Chen B Zhao Y : Data security and privacy-preserving in edge computing paradigm: Survey and open issues. *IEEE Access.* 2018;6:18209–18237. 10.1109/ACCESS.2018.2820162

[29] Tawalbeh LA Muheidat F Tawalbeh M : IoT Privacy and security: Challenges and solutions. *App. Sci.* 2020;10(12):4102. 10.3390/app10124102

[30] Sethi P Sarangi SR : Internet of things: architectures, protocols, and applications. *J. Elect. Com. Eng.* 2017;2017:1–25. 10.1155/2017/9324035

[31] El-Sayed H Chaqfeh M : Exploiting mobile edge computing for enhancing vehicular applications in smart cities. *Sensors.* 2019;19(5):1073. 10.3390/s19051073 30832386 PMC6427419

[32] Schroten A Van Grinsven A Tol E : The impact of emerging technologies on the transport system. 2020.

[33] Zhang B Zhang G Ma S : Efficient Multitask Scheduling for Completion Time Minimization in UAV-Assisted Mobile Edge Computing. *Mob. Inf. Syst.* 2020;2020:1–11. 10.1155/2020/8791030

[34] Mozaffari M Saad W Bennis M : A tutorial on UAVs for wireless networks: Applications, challenges, and open problems. *IEEE communications surveys & tutorials.* 2019;21(3):2334–2360. 10.1109/COMST.2019.2902862

[35] Outay F Mengash HA Adnan M : Applications of unmanned aerial vehicle (UAV) in road safety, traffic and highway infrastructure management: Recent advances and challenges. *Transp. Res. Part A Policy Pract.* 2020;141:116–129. 10.1016/j.tra.2020.09.018 33024357 PMC7527789

[36] Raza S Wang S Ahmed M : A survey on vehicular edge computing: architecture, applications, technical issues, and future directions. *Wirel. Commun. Mob. Comput.* 2019;2019:1–19. 10.1155/2019/3159762

[37] Ku YJ Chiang PH Dey S : Quality of service optimisation for vehicular edge computing with solar-powered road side units. *2018 27th International Conference on Computer Communication and Networks (ICCCN).* 2018, July; (pp.1–10). IEEE.

[38] Long J Luo Y Zhu X : Computation offloading through mobile vehicles in IoT-edge-cloud network. *EURASIP J. Wirel. Commun. Netw.* 2020;2020(1):1–21. 10.1186/s13638-020-01848-5

[39] Yu W Liang F He X : A survey on the edge computing for the Internet of Things. *IEEE Access.* 2017;6:6900–6919. 10.1109/ACCESS.2017.2778504

[40] Helfert M Klein C Donnellan B , editors. Smart Cities, Green Technologies and Intelligent Transport Systems: 8th International Conference, SMARTGREENS 2019, and 5th International Conference, VEHITS 2019, Heraklion, Crete, Greece, May 3-5, 2019, Revised Selected Papers (Vol. 1217).Springer Nature.2021.

