# Edge computing for Vehicle to Everything: a short review

**DOI:** 10.12688/f1000research.73269.3

**Published:** 2023-05-11

**Authors:** Mohd. Fikri Azli Abdullah, Sumendra Yogarayan, Siti Fatimah Abdul Razak, Afizan Azman, Anang Hudaya Muhamad Amin, Mazrah Salleh

**Affiliations:** 1 Faculty of Information Science and Technology, Multimedia University, Ayer Keroh, Melaka, 75450, Malaysia; 2 Faculty of Innovation & Technology, School of Computer Science, Taylors University, Subang, Java, 47500, Selangor, Malaysia; 3 Research and Innovation, Kolej Universiti Islam Melaka, Melaka, 78200, Malaysia; 4 Faculty of Computer, Information Science and Applied Media, Higher Colleges of Technology, Dubai, United Arab Emirates; 5 Civil Aero Data and Information, Rolls-Royce, Derby, England, UK

**Keywords:** V2X, Edge Computing, Review

## Abstract

Vehicle to Everything (V2X) communications and services have sparked considerable interest as a potential component of future Intelligent Transportation Systems. V2X serves to organise communication and interaction between vehicle to vehicle (V2V), vehicle to infrastructure (V2I), vehicle to pedestrians (V2P), and vehicle to networks (V2N). However, having multiple communication channels can generate a vast amount of data for processing and distribution. In addition, V2X services may be subject to performance requirements relating to dynamic handover and low latency communication channels. Good throughput, lower delay, and reliable packet delivery are the core requirements for V2X services.  Edge Computing (EC) may be a feasible option to address the challenge of dynamic handover and low latency to allow V2X information to be transmitted across vehicles. Currently, existing comparative studies do not cover the applicability of EC for V2X. This review explores EC approaches to determine the relevance for V2X communication and services. EC allows devices to carry out part or all of the data processing at the point where data is collected. The emphasis of this review is on several methods identified in the literature for implementing effective EC. We describe each method individually and compare them according to their applicability. The findings of this work indicate that most methods can simulate the EC positioning under predefined scenarios. These include the use of Mobile Edge Computing, Cloudlet, and Fog Computing. However, since most studies are carried out using simulation tools, there is a potential limitation in that crucial data in the search for EC positioning may be overlooked and ignored for bandwidth reduction. The EC approaches considered in this work are limited to the literature on the successful implementation of V2X communication and services. The outcome of this work could considerably help other researchers better characterise EC applicability for V2X communications and services.

## Introduction

**Figure 1.**
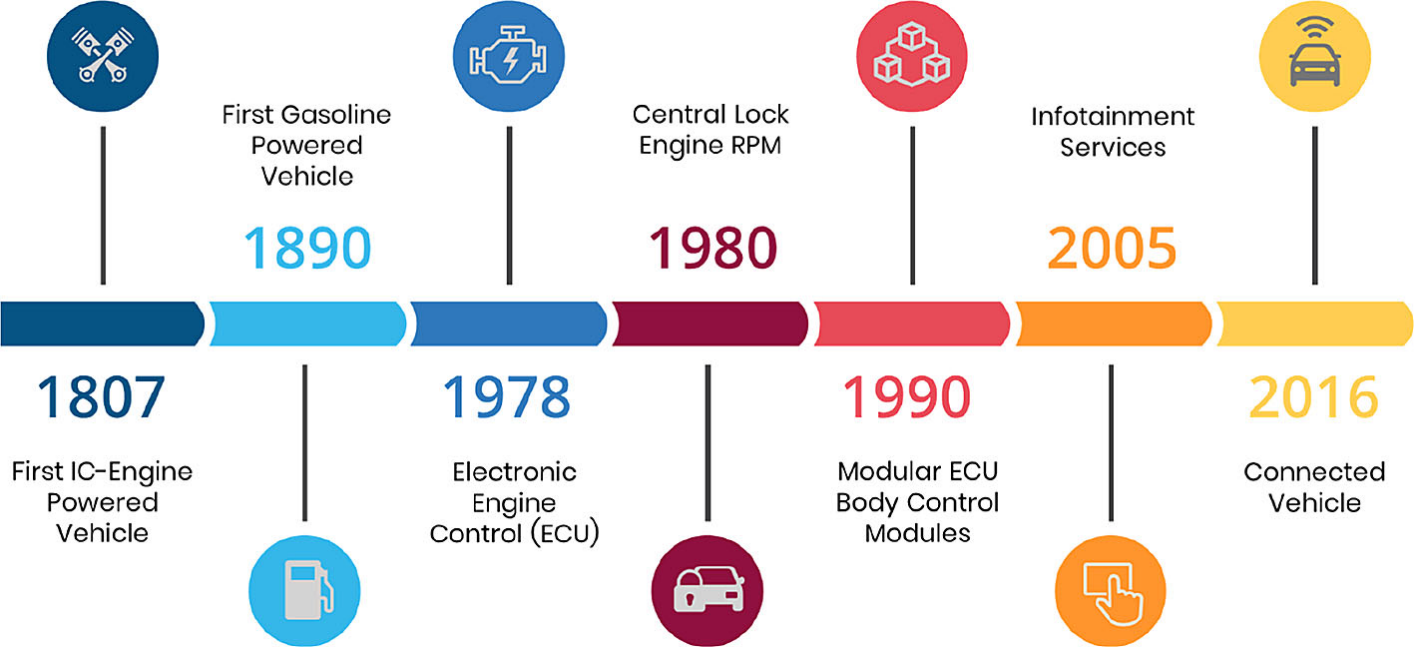
Evolution of driver and passenger interactions.

The automobile industry is changing in various ways, and this provides a chance to address potential transportation-related difficulties. This includes transitioning a traditional independent network to a connected network within and outside the vehicle. ^1^ The evolution of drivers' and passengers' involvement with vehicles has been evolving both in technology and style. ^2^ Figure 1 depicts the evolution of the interaction between drivers and passengers from 1807 to the present.

Almost everyone is connected to the Internet, with around six connected devices per person and hundreds of new connections created each second, resulting in billions of connected ecosystems. ^3^ Furthermore, research has projected that by 2025, connected vehicles will produce over 200 petabytes of data, with at least four terabytes of data generated continuously. This would increase the number of connected vehicles on roads by approximately four hundred million. ^4^ A connected vehicle is one that is equipped with both Internet and wireless LAN connectivity, allowing data to be transmitted between devices both inside and outside of the vehicle. The Internet of Vehicles or Vehicle to Everything (V2X) is a common network for connected vehicles. ^5^^,^^6^ However, the most challenging problem is efficiently processing and sending enormous data over communication networks.

The difficulty is not just handling data produced by these connected vehicles that are constantly exposed but also maintaining security, deployment, and performance. ^7^^,^^8^ Therefore, the potential of edge computing (EC) for V2X can play a prominent part. EC is a distributed computer system that carries out computational tasks (such as collecting and analysing data) on a device, particularly a vehicle. In turn, this reduces the transmission of data from the cloud back and forth. ^9^^,^^10^

The prospects of V2X and EC has significant potential for enabling a wide range of innovative applications and services for connected and autonomous vehicles. One of the main advantages of EC is its ability to enable real-time decision making for autonomous vehicles. ^2^^,^^3^ This includes tasks such as detecting and responding to obstacles or hazardous road conditions. By processing sensor data in real-time at the edge of the network, autonomous vehicles can make split-second decisions that enhance safety and efficiency. ^6^ In addition to enhancing safety, EC can also be used to provide personalized infotainment and other services to passengers based on their preferences and location. By analyzing data from a variety of sources, including social media, weather, and traffic patterns, EC can provide passengers with tailored recommendations for entertainment, food, and other services based on their location and preferences. ^9^^,^^10^ This can enhance the overall passenger experience and help differentiate connected and autonomous vehicles from traditional modes of transportation. This review examines EC, in particular for V2X. We discuss the background of automotive evolution, V2X and EC; prior research on the applicability of EC for V2X; the potential challenges of applying EC to the V2X scenario; and the path for the future.

## Automotive evolution, V2X, and EC

V2X communication is a crucial component of current intelligent transportation systems (ITS). For example, V2X provides drivers with information about road hazards that they may overlook. ^11^ In addition, V2X allows communication between a vehicle and anything that might impact the environment, including the surrounding infrastructure such as traffic lights (infrastructure) and even smartphones (pedestrian), enabling communication between vehicles and pedestrians holding a smartphone. ^12^ With the technology progressing globally, it is just a matter of time until it is widely adopted and deployed. ^13^

EC refers to a technology that allows network-level processing, downstream data for cloud services, and upstream data for IoT service support. ^14^ The term "edge" refers to any computer device located in the area between data sources and the cloud. EC is more suitable for applications that require rapid and consistent response times. ^15^ V2X is an example, as computing at the edge can reduce data transfer, decreasing reaction times. ^16^ For example, when driving, the vehicle captures data via movement, speed, and other sensors, then analyses them to ensure safety and convenience.

For V2X, real-time situational awareness is crucial, particularly on crucial route segments (e.g., an accident is detected by another vehicle on a particular road). Additionally, a backend server will have to provide high-definition local maps. Leveraging local maps and situational awareness is not just about providing data about road traffic conditions. It should also be extended to occurrences where local data must be aggregated in real-time and distributed to drivers on the road through road side units (RSUs). Road users may build and maintain real-time situational awareness using broadcast information from neighbour vehicles as an alternative to EC. Therefore, EC deployment enables shifting such activities to the network edge by combining data from many sources and efficiently broadcasting a huge amount of data to many drivers locally.

EC connects the processing and interpretation of inputs in the direct range to the end devices. The edge is a point of contact between vehicles and the cloud. The edge server has computing and storage means placed close to vehicles. ^17^ Therefore, the services furnished by edge computing produce a substantial level of quality of service to the end user (edge node). The current applications of in-vehicle networks should be supported by efficient communication and computational application. Edge servers analyse and stores the data acquired by the sensors in vehicles. Low-latency communication is made possible by these services, which also serve to promote awareness. ^18^^,^^19^

Software Defined Networking (SDN) is characterised by the independence of the data plane and control plane. The connection between the data plane and control plane causes packet flow to be delayed, which increases both the cost and the time required for transmission. ^20^ Thus, this has led the SDN to be concerned about reducing the amount of communication between the data plane and the control plane. The controller can use the real-time central global information provided by the network infrastructure to make informed management decisions. Through the use of a control plane, the controller has access to a network view. ^21^^,^^22^ It communicates with forwarding devices and offers network control applications with an integrated development environment. As a result, it helps the dynamic adoption and administration of networks without impacting the operations of existing network infrastructures. It helps in the development of new applications and efficient use of resources. The configuration of the SDN control plane enables the network to adapt to changes in network dynamics caused by unforeseen situations, such as traffic accidents or lane diversions. The SDN works in coordination with edge computing and supports the transportation systems’ ability to engage with the uncertainty of a varying and large number of vehicles, massive data volumes, and frequent disconnections to enable immediate vehicle connectivity and thus enhance the safety of the driver. ^23^^–^^25^

The architecture of EC is composed of three layers: cloud layer, edge cloud layer, and the vehicle network. A number of essential advantages of the cloud layer encompass batch processing, predictive analytics, analytical modeling, caching, high scalability, and data processing of detailed information, which are all far beyond the computing capabilities of the edge devices. ^26^ The cloud can process large volumes of data in a short time. Cloud infrastructure includes storage and processing. The data collected among many edge nodes will be transmitted to the cloud layer, entirely stored for long-term analysis. Since the data may be utilised over a more extended period, it would not be necessary to do computations in real-time. Components for computing and analysis conduct complicated computations more quickly. Unlike latency-sensitive services, computational processes transmitted suffer from delay. ^27^

On the other hand, the edge cloud layer links the vehicle network and the cloud layer. In order to do this, the vehicles are equipped with communication hardware that makes use of wireless communication protocols such as wireless LAN (IEEE 802.11n), dedicated short-range communication (IEEE 802.11p), cellular networks (4G/5G), and LoRa to communicate with one another and with their surroundings. ^28^^,^^29^ The purpose would be to provide reliable communication, emergency services, situational awareness, content identification, storage, processing, and increasing efficiency because it is situated close to vehicles and is used for reliable information. The edge cloud layer is beneficial for applications that need a faster response with very low delay, such as environment detection (flash flood), health recognition (drivers’ health condition), and driving behaviour identification. ^30^^,^^31^

Vehicles are expected to conduct more services. The vehicle network layer includes a collection of topologically nearby vehicles and shares computational and storage capabilities over a wireless network. ^32^ The vehicle network abstracts information from an on-board unit (OBU), a global positioning system (GPS), embedded sensors, cameras, lidar, radar, and other devices. The information obtained may be transmitted to the edge cloud layer for storing or utilised as a source for services offered at the application layer. As part of the vehicle network, the vehicle will be equipped with communications, intelligence, storage, and adapting capabilities that will allow it to expect the driver’s expectations. Additionally, the concept will also assist by offering offloading and computational capabilities for vehicles delivering all preferred services at the edge network. ^33^^,^^34^

## Applicability of EC for V2X

As of recent years, V2X and EC technologies are still in the early stages of development and deployment. However, there have been significant advancements in recent years, and many researchers and industry experts are optimistic about the future of these technologies. ^28^^,^^31^ In terms of V2X, many automakers and technology companies are investing in the development of connected and autonomous vehicles. In addition, several standards organizations, such as the IEEE and the 3GPP, are working to establish common standards and protocols for V2X communication. These standards will help ensure interoperability between different vehicles and systems and enable the development of a robust V2X ecosystem. ^29^^,^^30^ As for EC, there has been a rapid expansion of edge devices and infrastructure, driven by the increasing demand for low-latency and high-bandwidth applications such as autonomous driving and smart cities. Many cloud providers, such as Amazon Web Services and Microsoft Azure, are also expanding their edge computing capabilities to meet the growing demand for edge services. Moreover, there are several open-source projects and industry initiatives, such as the Open Edge Computing Initiative, aimed at promoting the development and adoption of EC technologies.

EC applies to a wide range of uses, from sensor applications (e.g., predictive vehicle maintenance) to the end-user experience (e.g., collision prevention warning). EC has been discussed previously from the perspective of V2X communication applicability. In 2020, Moubayed *et al.* described an Optimum V2X Service Placement (OVSP) as a binary integer issue in a linear edge context. ^35^ The authors approached this problem using a low-complexity greedy heuristic technique (G-VSPA). Extensive simulations showed that the OVSP model provides satisfactory results when sensitive services to delays are on the edge and tolerant services to delays are at the core of the process. Furthermore, the proposed algorithm provides near-optimal performance with minimal complexity.

In the same year, Shaer *et al*. addressed the efficient deployment of V2X essential services, including various V2X applications in the EC environment. ^36^ The authors devised an optimisation method for minimising E2E latency in multi-component V2V systems under different traffic situations. The findings indicate that the methodology guarantees an adequate level of service and surpasses solutions developed in earlier studies using realistic scenarios. Additionally, Belogaev *et al*. investigated task offloading that minimises operating costs while adhering to the latency constraints imposed by different V2X applications, given the network architecture and resource allocation. ^37^ The authors designed a new CHAT algorithm based on linear programming and incorporated a greedy algorithm. In terms of total energy usage, the suggested method was compared with previous studies proposed algorithms. The assessment demonstrates that the proposed method considerably decreases energy usage while meeting the varied needs of V2X applications in all evaluated cases.

Lee *et al.* described an EC approach for minimising trip time at interconnected junctions. ^38^ The authors suggested a paradigm in which each RSU determines junction scheduling while the vehicles select their travel trajectory through dynamic control. Based on simulation results for optimum scheduling of linked junctions, the proposed framework significantly reduced overall travel time by up to 14.3%. Grammarikos and Cottis investigated the benefits of mobile edge computing (MEC) adopting V2X services linked to traffic efficiency and road safety. ^39^ A simulation model that represented a long-term evolution (LTE) system with basic MEC capabilities, such as packet routing, was investigated in this work to evaluate the applicability of their findings. The presented approach evaluated the packet delivery ratio and packet loss for applications, such as telemetry and emergency message delivery, respectively. While LTE can transmit traffic data to vehicles in a short amount of time, the simulation results revealed that severe congestion in the backhaul and core networks could result in unexpected packet losses, which could be prevented by the processing capabilities of a MEC server.

In addition, Napolitano *et al.* proposed a fully compatible design and implementation of a vulnerable road users (VRU) warning system, as well as an experimental assessment of the system using MEC- and cloud-based architectures. ^40^ The authors developed a strategy that would enable road users to communicate information regarding the existence of neighbouring entities in the event of a difficult circumstance (e.g., road accident). This is accomplished by using an architecture that consists of a user-facing Android application and a MEC-based application [cooperative awareness messages (CAM)]. The E2E latency demonstrated a substantial result when visualising the entities engaged between the VRUs application and the CAM server using a preliminary performance measurement. Additionally, Emara *et al.* focused on the case of VRU, examining the safe interaction of vehicles with road users such as motorcyclists and pedestrians. ^41^ The authors aimed to describe latency improvements using MEC systems through periodic CAM. Extensive simulation results indicated that installing MEC infrastructure may substantially decrease the communication latency. Additionally, Sabella *et al.* suggested a hierarchical MEC architecture for adaptive video streaming in V2X applications. ^42^ The authors described the acquisition of real-time channel data by local agents stationed at the evolved NodeB (eNB). This information is then communicated to a MEC platform, which automatically changes the video stream's quality to match the channel's conditions. Within a virtualized network context, the authors tested and evaluated a conceptual demonstration of radio-aware video optimization. The results demonstrated that the proposed architecture enhanced the user experience by boosting downlink and uplink speeds and reducing delay.

Bissmeyer *et al*. introduced a network framework that ensures V2X information and data exchange in a MEC-based multi-access technology environment. ^43^ The authors designed a framework for the integrity of the message, sender authorisation and authentication, and replay detection. This approach is achieved through digital signatures, an authorisation certificate, and public and private key infrastructure. MEC offers local processing capabilities for the exchange of event-driven V2X encrypted messages within the framework. In addition, Balid *et al.* demonstrated MEC traffic management methods for real-time traffic monitoring. ^44^ The authors developed and deployed a cost-effective wireless sensor traffic monitoring system for highway and roadside traffic. The sensor achieved an acceptable level of accuracy in terms of detection, speed prediction, and vehicle categorisation.

## Challenges of V2X and EC

### Security

At the edge of a network, privacy and security protection are critical services to provide. ^45^^,^^46^ If the vehicle is equipped with IoT, it can collect sensitive data from sense data. ^47^ Several ITS implementations would need drivers to grant access to sensitive, confidential data to untrusted vehicles attempting to join as edges in the context of smart cities. ^48^ Together with data segregation techniques, effective trust management systems may considerably increase edge security. ^49^ According to El-Sayed & Chaqfeh, ^50^ although minimal research has been conducted on assuring secure collaboration in an EC scenario, the study does not explicitly address V2X issues.

### Deployments

The positioning of edge devices in an urban environment is based on static and dynamic features. ^51^ Edge nodes may need MEC servers with fixed RSUs or unmanned aerial vehicles (UAV). ^52^ Many possible ITS applications may be facilitated by autonomous UAVs, improving traffic safety and transportation quality of life. ^53^ Nevertheless, specific issues must be addressed, such as limited energy, processing ability, and signal transmission range. ^54^ Given the technological developments such as sensor-based street lights or smart toll booths over the past few decades, the limitations on UAV usage will likely be overcome eventually.

### Performances

Each second counts when you're behind the wheel of a vehicle. As a result, vehicles would continuously upload the data collected by their local sensors to the closest edge device. ^55^^,^^56^ Hence, energy and power consumption at the edge should be considered to avoid service disruptions and quality of service (QoS) loss. ^57^^,^^58^ Furthermore, various situations need substantial QoS improvement to cope with occasional high traffic loads like severe traffic congestion, unpredicted weather conditions, or unexpected road construction works. ^59^ Therefore, further research is necessary to enhance and manage QoS in the V2X context considering a heterogeneous edge-based environment.

## Conclusions

EC adoption is growing in the automotive industry, and ITS, particularly V2X, will certainly change various economic sectors and significantly influence our everyday lives. Despite this, multiple different challenges are limiting its wide implementation. The increasing number of sensors in connected vehicles and roads creates a large data processing and storage issue. This requires new service platforms with strong processing, reliable storage, and real-time communication. EC is indeed a promising way to decrease latency and bring data closer to vehicles and resources. In the future, we will work on a comprehensive middleware solution for V2X communication. In many V2X scenarios, data transmitted between users and network infrastructure is localised and does not need remote access to centralised data centres. Using EC may substantially improve the performance of supporting various applications of V2X. The availability of network resources, storage, and computation near the network edge make EC an ideal option for V2X delay-sensitive applications.

## Data availability

No data is associated with this article.

## References

[1] Kirkland G : How new technologies have changed the automotive industry. 2019. Reference Source

[2] Shurpali S : Role of Edge Computing in Connected and Autonomous Vehicles. 2020. Reference Source

[3] Statista: Number of Internet of things (IoT) connected devices worldwide in 2018, 2025 and 2030. 2019. Reference Source

[4] Pati VP : Edge Insights for Superior Autonomous Vehicle Experience. 2020. Reference Source

[5] Uhlemann E : Introducing connected vehicles [connected vehicles]. *IEEE Vehicular Technology Magazine.* 2015;10(1):23–31. 10.1109/MVT.2015.2390920

[6] Coppola R Morisio M : Connected car: technologies, issues, future trends. *ACM Computing Surveys (CSUR).* 2016;49(3):1–36. 10.1145/2971482

[7] Guerrero-Ibáñez J Zeadally S Contreras-Castillo J : Sensor technologies for intelligent transportation systems. *Sensors.* 2018;18(4):1212. 10.3390/s18041212 29659524 PMC5948625

[8] Giust F Sciancalepore V Sabella D : Multi-access edge computing: The driver behind the wheel of 5G-connected cars. *IEEE Communications Standards Magazine.* 2018;2(3):66–73. 10.1109/MCOMSTD.2018.1800013

[9] Ai Y Peng M Zhang K : Edge computing technologies for Internet of Things: a primer. *Digital Communications and Networks.* 2018;4(2):77–86. 10.1016/j.dcan.2017.07.001

[10] Yousefpour A Fung C Nguyen T : All one needs to know about fog computing and related edge computing paradigms: A complete survey. *Journal of Systems Architecture.* 2019;98:289–330. 10.1016/j.sysarc.2019.02.009

[11] Kiela K Barzdenas V Jurgo M : Review of V2X–IoT standards and frameworks for ITS applications. *Applied Sciences.* 2020;10(12):4314. 10.3390/app10124314

[12] Naranjo JE Jiménez F Anaya JJ : Application of vehicle to another entity (V2X) communications for motorcycle crash avoidance. *Journal of Intelligent Transportation Systems.* 2017;21(4):285–295. 10.1080/15472450.2016.1247703

[13] Ahlborn B : Five Reasons Why We Benefit from V2X. 2016. Reference Source

[14] Sittón-Candanedo I Corchado JM : An Edge Computing Tutorial. *Oriental Journal of Computer Science and Technology.* 2019;12(2):34–38. 10.13005/ojcst12.02.02

[15] Shi W Dustdar S : The promise of edge computing. *Computer.* 2016;49(5):78–81. 10.1109/MC.2016.145

[16] Weisong S Xingzhou Z Yifan W : Edge computing: state-of-the-art and future directions. *Journal of Computer Research and Development.* 2019;56(1):69.

[17] Feng J Liu Z Wu C : AVE: Autonomous vehicular edge computing framework with ACO-based scheduling. *IEEE Transactions on Vehicular Technology.* 2017;66(12):10660–10675. 10.1109/TVT.2017.2714704

[18] Hou X Li Y Chen M : Vehicular fog computing: a viewpoint of vehicles as the infrastructures. *IEEE Transactions on Vehicular Technology.* 2016;65(6):3860–3873. 10.1109/TVT.2016.2532863

[19] Liu L Chen C Pei Q : Vehicular edge computing and networking: A survey. *Mobile Networks and Applications.* 2021;26(3):1145–1168. 10.1007/s11036-020-01624-1

[20] Karakus M Durresi A : A survey: Control plane scalability issues and approaches in software-defined networking (SDN). *Computer Networks.* 2017;112:279–293. 10.1016/j.comnet.2016.11.017

[21] Li H Dong M Ota K : Control Plane Optimization in Software-Defined Vehicular Ad Hoc Networks. *IEEE Transactions on Vehicular Technology.* 2016;65(10):7895–7904. 10.1109/TVT.2016.2563164

[22] Kreutz D Ramos FMV Verissimo PE : Software-defined networking: a comprehensive survey. *Proceedings of the IEEE.* 2015;103(1):14–76. 10.1109/JPROC.2014.2371999

[23] Kai K Cong W Tao L : Fog computing for vehicular Ad-hoc networks: paradigms, scenarios, and issues. *Journal of China Universities of Posts and Telecommunications.* 2016;23(2):56–96. 10.1016/S1005-8885(16)60021-3

[24] Lee E-K Gerla M Pau G : Internet of Vehicles: From intelligent grid to autonomous cars and vehicular fogs. *International Journal of Distributed Sensor Networks.* 2016;12(9):155014771666550. 10.1177/1550147716665500

[25] El-Sayed H Sankar S Prasad M : Edge of things: The big picture on the integration of edge, IoT and the cloud in a distributed computing environment. *IEEE Access.* 2017;6:1706–1717.

[26] Chen CP Zhang CY : Data-intensive applications, challenges, techniques and technologies: A survey on Big Data. *Information Sciences.* 2014;275:314–347. 10.1016/j.ins.2014.01.015

[27] Ku I Lu Y Gerla M : Towards software-defined VANET: Architecture and services.In *Proceedings of the IEEE conference on Ad Hoc Networking Workshop*, pp.103–110,2014.

[28] Zhou H Xu W Chen J : Evolutionary V2X technologies toward the Internet of vehicles: Challenges and opportunities. *Proceedings of the IEEE.* 2018;108(2):308–323. 10.1109/JPROC.2019.2961937

[29] Wang J Liu J Kato N : Networking and communications in autonomous driving: A survey. *IEEE Communications Surveys & Tutorials.* 2018;21(2):1243–1274.

[30] Ni J Zhang K Lin X : Securing fog computing for internet of things applications: Challenges and solutions. *IEEE Communications Surveys & Tutorials.* 2017;20(1):601–628. 10.1109/COMST.2017.2762345

[31] Meneguette R De Grande R Ueyama J : Vehicular Edge Computing: Architecture, Resource Management, Security, and Challenges. *ACM Computing Surveys (CSUR).* 2021;55(1):1–46. 10.1145/3485129

[32] Chen M Tian Y Fortino G : Cognitive internet of vehicles. *Computer Communications.* 2018;120:58–70. 10.1016/j.comcom.2018.02.006

[33] Yuan Q Zhou H Li J : Toward efficient content delivery for automated driving services: An edge computing solution. *IEEE Network.* 2018;32(1):80–86. 10.1109/MNET.2018.1700105

[34] Zhang J Letaief KB : Mobile edge intelligence and computing for the internet of vehicles. *Proceedings of the IEEE.* 2019;108(2):246–261. 10.1109/JPROC.2019.2947490

[35] Moubayed A Shami A Heidari P : Edge-enabled V2X service placement for intelligent transportation systems. *IEEE Transactions on Mobile Computing.* 2020;20:1380–1392. 10.1109/TMC.2020.2965929

[36] Shaer I Haque A Shami A : Multi-Component V2X Applications Placement in Edge Computing Environment. *ICC 2020-2020 IEEE International Conference on Communications (ICC).* 2020; (pp.1–6).IEEE.

[37] Belogaev A Elokhin A Krasilov A : Cost-effective V2X task offloading in MEC-assisted intelligent transportation systems. *IEEE Access.* 2020;8:169010–169023. 10.1109/ACCESS.2020.3023263

[38] Lee G Guo J Kim KJ : Edge Computing for Interconnected Intersections in Internet of Vehicles. *2020 IEEE Intelligent Vehicles Symposium (IV).* 2020; (pp.480–486). IEEE.

[39] Grammatikos PV Cottis PG : A Mobile Edge Computing Approach for Vehicle to Everything Communications. *Communications and Network.* 2019;11(3):65–81. 10.4236/cn.2019.113006

[40] Napolitano A Cecchetti G Giannone F : Implementation of a MEC-based vulnerable road user warning system. *2019 AEIT International Conference of Electrical and Electronic Technologies for Automotive (AEIT AUTOMOTIVE).* 2019; (pp.1–6). IEEE.

[41] Emara M Filippou MC Sabella D : MEC-assisted end-to-end latency evaluations for C-V2X communications. *2018 European conference on networks and communications (EuCNC).* 2018; (pp.1–9). IEEE.

[42] Sabella D Nikaein N Huang A : A hierarchical MEC architecture: Experimenting the RAVEN use-case. *2018 IEEE 87th Vehicular Technology Conference (VTC Spring).* 2018; (pp.1–5). IEEE.

[43] Bissmeyer N Dam JF van Zimmermann C : Security in hybrid vehicular communication based on its-g5, lte-v, and mobile edge computing. *AmE 2018-Automotive meets Electronics; 9th GMM-Symposium.* 2018; (pp.1–6). VDE.

[44] Balid W Tafish H Refai HH : Intelligent vehicle counting and classification sensor for real-time traffic surveillance. *IEEE Transactions on Intelligent Transportation Systems.* 2017;19(6):1784–1794. 10.1109/TITS.2017.2741507

[45] Zhong S Zhong H Huang X : *Security and Privacy for Next-Generation Wireless Networks.* Springer International Publishing;2019.

[46] Zhang J Chen B Zhao Y : Data security and privacy-preserving in edge computing paradigm: Survey and open issues. *IEEE Access.* 2018;6:18209–18237. 10.1109/ACCESS.2018.2820162

[47] Tawalbeh LA Muheidat F Tawalbeh M : IoT Privacy and security: Challenges and solutions. *Applied Sciences.* 2020;10(12):4102. 10.3390/app10124102

[48] Sethi P Sarangi SR : Internet of things: architectures, protocols, and applications. *Journal of Electrical and Computer Engineering.* 2017;2017:1–25. 10.1155/2017/9324035

[49] El-Sayed H Chaqfeh M : Exploiting mobile edge computing for enhancing vehicular applications in smart cities. *Sensors.* 2019;19(5):1073. 10.3390/s19051073 30832386 PMC6427419

[50] Schroten A Van Grinsven A Tol E : The impact of emerging technologies on the transport system. 2020.

[51] Zhang B Zhang G Ma S : Efficient Multitask Scheduling for Completion Time Minimization in UAV-Assisted Mobile Edge Computing. *Mobile Information Systems.* 2020;2020:1–11. 10.1155/2020/8791030

[52] Mozaffari M Saad W Bennis M : A tutorial on UAVs for wireless networks: Applications, challenges, and open problems. *IEEE Communications Surveys & Tutorials.* 2019;21(3):2334–2360. 10.1109/COMST.2019.2902862

[53] Outay F Mengash HA Adnan M : Applications of unmanned aerial vehicle (UAV) in road safety, traffic and highway infrastructure management: Recent advances and challenges. *Transportation Research Part A: Policy and Practice.* 2020;141:116–129. 10.1016/j.tra.2020.09.018 33024357 PMC7527789

[54] Raza S Wang S Ahmed M : A survey on vehicular edge computing: architecture, applications, technical issues, and future directions. *Wireless Communications and Mobile Computing.* 2019;2019:1–19. 10.1155/2019/3159762

[55] Ku YJ Chiang PH Dey S : Quality of service optimisation for vehicular edge computing with solar-powered road side units. *2018 27th International Conference on Computer Communication and Networks (ICCCN).* 2018, July; (pp.1–10). IEEE.

[56] Long J Luo Y Zhu X : Computation offloading through mobile vehicles in IoT-edge-cloud network. *EURASIP Journal on Wireless Communications and Networking.* 2020;2020(1):1–21. 10.1186/s13638-020-01848-5

[57] Yu W Liang F He X : A survey on the edge computing for the Internet of Things. *IEEE Access.* 2017;6:6900–6919. 10.1109/ACCESS.2017.2778504

[58] Helfert M Klein C Donnellan B , editors. *Smart Cities, Green Technologies and Intelligent Transport Systems: 8th International Conference, SMARTGREENS 2019, and 5th International Conference, VEHITS 2019, Heraklion, Crete, Greece, May 3-5, 2019, Revised Selected Papers (Vol. 1217).*Springer Nature.2021.

[59] Boukerche A Robson E : Vehicular cloud computing: Architectures, applications, and mobility. *Computer Networks.* 2018;135:171–189.

