# Supplementary material for: Edge computing for Vehicle to Everything: a short review
Source: F1000Res. 2023 Nov 10;10:1104. Originally published 2021 Nov 1. [Version 4] doi: 10.12688/f1000research.73269.4 (PMC11002521; doi:10.12688/f1000research.73269.4)
Supplement: Peer review history

# Reviewer report: Reviewer response for version 4

**DOI:** 10.5256/f1000research.154335.r241475

**Published:** 2024-04-08

**Authors:** Preeti Rani

**Affiliations:** 1 SRM Institute of science and Technology,, Delhi-NCR Campus, India

The authors have presented a satisfied review vehicle to everything’s techniques. The manuscript may have some merits, but some major concerns are:

1. The presentation of the paper can be significantly improved if the authors review the paper with a language specialist and try to polish it in order to be more readable: such as long sentences, tense, misused punctuation, etc. Try to put some lined space in between each paragraph for better paper visualization.
2. The gap statement of the research is a little bit poor and insufficient and the main contributions of the authors need to be included in the introduction section.
3. The conclusion section includes only a summary of conclusions from the published studies, there are no findings of this review paper.
4. What is the motivation and mechanism behind the survey? The section is completely missing.
5. The abbreviations are repeatedly declared. It needs to be declared once then used as abbreviation only.
6. Authors need to add 2-3 comparatives tables.

Is the review written in accessible language?

Yes

Are all factual statements correct and adequately supported by citations?

Yes

Are the conclusions drawn appropriate in the context of the current research literature?

Yes

Is the topic of the review discussed comprehensively in the context of the current literature?

Yes

Reviewer Expertise:

Wireless Technology, Wireless Sensor Network, Networking, Wireless Computing, Satellite Communication, Network Simulation, Computer Networking, Network Communication, Routing Network,   QoS, Security Network, Mobile Ad-hoc Network, Vehicular Ad-hoc Network, Internet of Vehicle

I confirm that I have read this submission and believe that I have an appropriate level of expertise to confirm that it is of an acceptable scientific standard, however I have significant reservations, as outlined above.

# Reviewer report: Reviewer response for version 4

**DOI:** 10.5256/f1000research.154335.r241476

**Published:** 2024-03-06

**Authors:** Qiong Wu

**Affiliations:** 1 Jiangnan University, Wuxi, China

This article describes the evolution of V2X and EC technology and reviews the references on the new service technologies that are being created by the combination of the two technologies. However, the authors have not given the deep insight on these references, but simply listed them and thus it is lack of contributions. At the same time, the authors also listed the challenges and expectations that exist in the existing works, which are quite interesting to consider. My detailed comments are given as follows,

 Major comments

 1. "V2X encompasses two main technologies: C-V2X and DSRC" is mentioned in the article, but the application and comparison of the two technologies in the existing works on EC should be added. The authors have not addressed the issues raised in the previous revisions.

 2. The article mentions "We compare them(mobile edge computing, cloudlet, and fog computing) according to their applicability to V2X" in Abstract, however, there is only an introduction in the paper without comparison. As a review, the comparison among mobile edge computing, cloudlet, and fog computing should be given.

 3. From the Abstract, the paper focuses on EC as a promising solution to the challenge of V2X massive data processing, but does not presents the comparison of specific methods of data processing. It is more convincing to add horizontal comparisons between the related works to illustrate the performance differences and advantages and disadvantages of the existing methods under the same conditions.

 Minor comments

 This article is a review that lists some applications, but lacks relevant literature citations, e.g. references should be inserted after "predictive vehicle maintenance".

 1) [1]

 2) [3]

 3) [2]

 4) [4]

Is the review written in accessible language?

Yes

Are all factual statements correct and adequately supported by citations?

Partly

Are the conclusions drawn appropriate in the context of the current research literature?

Yes

Is the topic of the review discussed comprehensively in the context of the current literature?

Partly

Reviewer Expertise:

My research area belongs to image processing in self-driving, which involves the processing of a large amount of data, and has some things in common with the problem that the paper aims to illustrate.

I confirm that I have read this submission and believe that I have an appropriate level of expertise to state that I do not consider it to be of an acceptable scientific standard, for reasons outlined above.

## References

[1] : URLLC-Awared Resource Allocation for Heterogeneous Vehicular Edge Computing. *IEEE Transactions on Vehicular Technology* .2024; 10.1109/TVT.2024.3370196 1-16 10.1109/TVT.2024.3370196

[2] : Delay-Sensitive Task Offloading in Vehicular Fog Computing-Assisted Platoons. *IEEE Transactions on Network and Service Management* .2024; 10.1109/TNSM.2023.3322881 10.1109/TNSM.2023.3322881

[3] : Mobility-Aware Cooperative Caching in Vehicular Edge Computing Based on Asynchronous Federated and Deep Reinforcement Learning. *IEEE Journal of Selected Topics in Signal Processing* .2023;17(1) : 10.1109/JSTSP.2022.3221271 66-81 10.1109/JSTSP.2022.3221271

[4] : Towards V2I Age-Aware Fairness Access: A DQN Based Intelligent Vehicular Node Training and Test Method. *Chinese Journal of Electronics* .2023;32(6) : 10.23919/cje.2022.00.093 1230-1244 10.23919/cje.2022.00.093

# Reviewer report: Reviewer response for version 4

**DOI:** 10.5256/f1000research.154335.r241478

**Published:** 2024-02-21

**Authors:** Mahmood A Al-Shareeda

**Affiliations:** 1 Iraq University College, Basrah, Iraq

The title is timing and interested. There is sound research in the context of the manuscript. The following comments have been provided to improve the quality of the manuscript.

 1-add numerical result in the Abstract part.

 2-List of contribution should be added to the introduction part.

 3-Authors are recommended to add more figures to describe the proposed solution well.

 4-What is the difference between the proposal and other relevant research? And how is your proposal outperformed others?

 5-Reviewing and citing the following modern solutions in related work part:[1],[2],[3],[4],[5],[6],[7].

Is the review written in accessible language?

Yes

Are all factual statements correct and adequately supported by citations?

Yes

Are the conclusions drawn appropriate in the context of the current research literature?

Yes

Is the topic of the review discussed comprehensively in the context of the current literature?

Yes

Reviewer Expertise:

VANET, vehicle

I confirm that I have read this submission and believe that I have an appropriate level of expertise to confirm that it is of an acceptable scientific standard, however I have significant reservations, as outlined above.

## References

[1] : FCA-VBN: Fog computing-based authentication scheme for 5G-assisted vehicular blockchain network. *Internet of Things* .2024;25: 10.1016/j.iot.2024.101096 10.1016/j.iot.2024.101096

[2] : HAFC: Handover Authentication Scheme Based on Fog Computing for 5G-Assisted Vehicular Blockchain Networks. *IEEE Access* .2024;12: 10.1109/ACCESS.2024.3351278 6251-6261 10.1109/ACCESS.2024.3351278

[3] : A Novel DDoS Mitigation Strategy in 5G-Based Vehicular Networks Using Chebyshev Polynomials. *Arabian Journal for Science and Engineering* .2023; 10.1007/s13369-023-08535-9 10.1007/s13369-023-08535-9

[4] : COVID-19 Vehicle Based on an Efficient Mutual Authentication Scheme for 5G-Enabled Vehicular Fog Computing. *Int J Environ Res Public Health* .2022;19(23) : 10.3390/ijerph192315618 10.3390/ijerph192315618 36497709 PMC9740694

[5] : SE-CPPA: A Secure and Efficient Conditional Privacy-Preserving Authentication Scheme in Vehicular Ad-Hoc Networks. *Sensors (Basel)* .2021;21(24) : 10.3390/s21248206 10.3390/s21248206 34960311 PMC8706963

[6] : Towards Identity-Based Conditional Privacy-Preserving Authentication Scheme for Vehicular Ad Hoc Networks. *IEEE Access* .2021;9: 10.1109/ACCESS.2021.3104148 113226-113238 10.1109/ACCESS.2021.3104148

[7] : MSR-DoS: Modular Square Root-Based Scheme to Resist Denial of Service (DoS) Attacks in 5G-Enabled Vehicular Networks. *IEEE Access* .2022;10: 10.1109/ACCESS.2022.3222488 120606-120615 10.1109/ACCESS.2022.3222488

# Reviewer report: Reviewer response for version 3

**DOI:** 10.5256/f1000research.147679.r184319

**Published:** 2023-08-01

**Authors:** Muhammad Saleh Bute

**Affiliations:** 1 Southwest Jiaotong University, Chengdu, Sichuan, China

1. In the abstract, the aim of this work is not clear? Is it about placement of edge computing resources or a review on edge computing and V2X?
2. I think before going into proper review, it is good to look into edge computing standard set by the ETSI. Which is not stated in the work. secondly, V2X is also of two kinds, the C-V2X and DSRC, which are not explicitly discussed in the work.
3. Insufficient references for a review work
4. The authors, did not satisfactorily state the findings of the review nor stating a clear guide for future work/direction.

Is the review written in accessible language?

Yes

Are all factual statements correct and adequately supported by citations?

Partly

Are the conclusions drawn appropriate in the context of the current research literature?

No

Is the topic of the review discussed comprehensively in the context of the current literature?

Partly

Reviewer Expertise:

Vehicular Communications, Edge Computing

I confirm that I have read this submission and believe that I have an appropriate level of expertise to state that I do not consider it to be of an acceptable scientific standard, for reasons outlined above.

# Reviewer report: Reviewer response for version 2

**DOI:** 10.5256/f1000research.135574.r143530

**Published:** 2023-03-02

**Authors:** Danilo Amendola

**Affiliations:** 1 Vicomtech, Donostia-San Sebastiá, Spain; 2 University of Trieste, Trieste, Italy

The work has not been sufficiently improved to achieve a better approval status, as the authors have not address the concerns raised in the peer review report for the first version of the article.

Is the review written in accessible language?

Yes

Are all factual statements correct and adequately supported by citations?

Yes

Are the conclusions drawn appropriate in the context of the current research literature?

Partly

Is the topic of the review discussed comprehensively in the context of the current literature?

Yes

Reviewer Expertise:

NA

I confirm that I have read this submission and believe that I have an appropriate level of expertise to state that I do not consider it to be of an acceptable scientific standard, for reasons outlined above.

# Reviewer report: Reviewer response for version 2

**DOI:** 10.5256/f1000research.135574.r143531

**Published:** 2022-07-18

**Authors:** Lionel Nkenyereye

**Affiliations:** 1 Research Institute of Engineering, Pukyong National University, Busan, South Korea

The authors have successfully addressed my concerns.

Is the review written in accessible language?

Yes

Are all factual statements correct and adequately supported by citations?

Yes

Are the conclusions drawn appropriate in the context of the current research literature?

Yes

Is the topic of the review discussed comprehensively in the context of the current literature?

Yes

Reviewer Expertise:

NA

I confirm that I have read this submission and believe that I have an appropriate level of expertise to confirm that it is of an acceptable scientific standard.

# Reviewer report: Reviewer response for version 1

**DOI:** 10.5256/f1000research.76911.r123882

**Published:** 2022-03-02

**Authors:** Lionel Nkenyereye

**Affiliations:** 1 Research Institute of Engineering, Pukyong National University, Busan, South Korea

The paper discussed the successful implementation of V2X communication and services. The V2X services coupled with edge computing prompt new V2X services with low latency. The authors have provided use cases that show the effectiveness of deploying edge computing for V2X-based edge computing applications. 

 The paper is well written and organized. The following are some comments that should be addressed:

1. The topic of edge computing supported for V2X is wide and there are interesting concepts which the authors have not yet discussed such as the task offloading (vehicle -to edge servers), edge caching to cache the data in edge to reduce communication latency. 
2. The paper is well written. Since it is a short review, it is clear that this article lacks new contributions. I would like to ask the authors what the contributions are.
3. Regarding the implementation of V2X communications: How is this implementation achieved?

Is the review written in accessible language?

Yes

Are all factual statements correct and adequately supported by citations?

Yes

Are the conclusions drawn appropriate in the context of the current research literature?

Yes

Is the topic of the review discussed comprehensively in the context of the current literature?

Yes

Reviewer Expertise:

Edge computing, vehicle network, V2X, Internet of things

I confirm that I have read this submission and believe that I have an appropriate level of expertise to confirm that it is of an acceptable scientific standard, however I have significant reservations, as outlined above.

# Response

**Published:** 2022-06-26

**Authors:** Sumendra Yogarayan

**Affiliations:** Multimedia University, Malaysia

The paper has been revised accordingly.

 In regards to comment number 2, the study contributes to the domain of edge computing incorporation for vehicular communication particularly in Malaysia context.

 In regards to comment number 3, as of recent, the national automotive policy 2020 has initiated the standard for connected vehicles that includes on vehicle to vehicle communication. Thus, this certainly will add to the understanding of edge computing for further integration in safety related or non-related safety application.

# Reviewer report: Reviewer response for version 1

**DOI:** 10.5256/f1000research.76911.r98480

**Published:** 2021-12-20

**Authors:** Danilo Amendola

**Affiliations:** 1 Vicomtech, Donostia-San Sebastiá, Spain; 2 University of Trieste, Trieste, Italy

The article presents in a nutshell a short, partial, and shallow overview of edge computing.

 The authors introduce the historic evolution of driver and passengers' interactions since the dawn, however the contribution related to the vehicular communications and edge computing in the paper is not enough wide. 

 There are no references to the standards, architecture, and challenges about the edge computing. The contribution is poor, and the related works considered are too few to consider the work valid.

 The author should extend the paper with more related works and deep analysis about the status of technology on V2X and edge computing.

Is the review written in accessible language?

Yes

Are all factual statements correct and adequately supported by citations?

Yes

Are the conclusions drawn appropriate in the context of the current research literature?

Partly

Is the topic of the review discussed comprehensively in the context of the current literature?

Yes

Reviewer Expertise:

NA

I confirm that I have read this submission and believe that I have an appropriate level of expertise to state that I do not consider it to be of an acceptable scientific standard, for reasons outlined above.

## References

[1] : Reliable Adaptive Resource Management for Cognitive Cloud Vehicular Networks. *IEEE Transactions on Vehicular Technology* .2015;64(6) : 10.1109/TVT.2014.2345767 2528-2537 10.1109/TVT.2014.2345767

# Response

**Published:** 2022-06-26

**Authors:** Sumendra Yogarayan

**Affiliations:** Multimedia University, Malaysia

The paper has been revised accordingly.
